# Empathy and Equity: Key Considerations for Large Language Model Adoption in Health Care

**DOI:** 10.2196/51199

**Published:** 2023-12-28

**Authors:** Erica Koranteng, Arya Rao, Efren Flores, Michael Lev, Adam Landman, Keith Dreyer, Marc Succi

**Affiliations:** 1 Harvard Medical School Boston, MA United States; 2 Massachusetts General Hospital Boston United States

**Keywords:** ChatGPT, AI, artificial intelligence, large language models, LLMs, ethics, empathy, equity, bias, language model, health care application, patient care, care, development, framework, model, ethical implication

## Abstract

The growing presence of large language models (LLMs) in health care applications holds significant promise for innovative advancements in patient care. However, concerns about ethical implications and potential biases have been raised by various stakeholders. Here, we evaluate the ethics of LLMs in medicine along 2 key axes: empathy and equity. We outline the importance of these factors in novel models of care and develop frameworks for addressing these alongside LLM deployment.

## Introduction

The rapid proliferation of applications that leverage the ability of large language models (LLMs) to use large amounts of complex information to find relevant patterns and apply them to novel use cases promises great innovation in health care and many other sectors. Many health care applications, such as clinical decision support, patient education, electronic health records (EHRs), and workflow optimization, have been proposed [1]. Despite the immense potential advantages of this technology, various key stakeholders have raised concerns regarding its ethical implications and potential perpetuation of existing biases and structural barriers [2-6]. Furthermore, its growing usage in the health care setting also raises the concern of transparency or disclosure about its use and role in patient management. Ethically incorporating LLMs into health care delivery requires honest dialogue about the principles we aim to uphold in patient care and a comprehensive analysis of the various ways in which LLMs could bolster or impair these.

Studies have demonstrated the utility of LLMs as a clinical decision support tool in various settings, including in triage, diagnostics, and treatment [7-11]. While LLMs show great promise in improving the efficiency of clinical workflows, they lack one key facet of physician-patient encounters: empathy. Though LLMs can be trained to use empathetic language [12] and have been able to use empathetic language in patient interactions [13], this concept of artificial empathy is easily distinguishable from real empathy from a patient’s perspective, and real empathy matters to patients [14]. The concept of artificial empathy, which aims to imbue artificial intelligence (AI) with human-like empathy, ought not to be considered interchangeable with human empathy. Efforts made to design artificial empathy, while commendable, should aim to be complementary to human empathy in order to avoid further isolating patients in their time of need by destroying the therapeutic alliance between patients and physicians [15]. Loneliness is one of the key public health crises of our time, and conflating technology with human-to-human interaction will only exacerbate this [16]. Empathic care for patients should be one of the core mandates of the health care sector, and true empathy requires human connection. Therefore, while LLMs show great promise in clinical workflows, they should augment, rather than replace, physician-led care (Table 1).

In addition to empathy, equity is crucial in novel models of care. The current most popular LLMs, including ChatGPT, Bard, Med-PaLM, and others, are trained on vast sources of data, including wide swaths of the internet. These sources are rife with inherent biases and lack transparency regarding the contents of the training data sets. They also lack specific evaluation of model biases, which may be harbingers of ethical dilemmas via the rapid incorporation of LLMs into clinical spaces. While there is little consensus regarding the degree of bias in current LLMs, in most embedding models, which have similar underlying architecture, there is evidence of racial, gender, and age bias [17]. LLMs have been demonstrated to associate negative terms with given names that are popular among the African American as well as with the masculine poles of most gender axes [17]. Until systematic evaluation of LLMs is performed in clinical use cases to understand and mitigate biases against vulnerable demographics, careful risk-benefit calculations and a regulatory framework should be implemented by relevant governing bodies before LLMs are permitted in clinical care. This framework must ensure that these models are improving health care delivery and outcomes for all. Importantly, the US Food and Drug Administration lacks a robust authorization pathway for software as a medical device; this in itself is challenging, and given the rapid development of LLMs, would benefit from expeditious guidelines [18] (see Table 2 for proactive measures to ensure the equitable incorporation of LLMs into health care). Following a previously published ethical framework for integrating innovative domains into medicine, we suggest an LLM framework guided by Blythe et al [19] grounded in principled primary motivations as detailed in Tables 1 and 2.

Despite these ethical risks, the potential benefits of incorporating LLMs into health care are numerous. LLMs are adept at quickly synthesizing large amounts of complex data, which can form the basis for numerous applications in the health care sector, including the management and interpretation of EHRs and clinical notes, adjuncts for patient visits (eg, encounter transcription and patient translation), billing for medical services, patient education, and more [20,21]. Thus, the key ethical question at hand is as follows: do the benefits outweigh the risks?

From a utilitarian perspective, we must consider this question to not only enhance decision-making but also take advantage of opportunities to mitigate potential harms. Proposals for the incorporation of a systematized, frequently reevaluated method of bias evaluation into clinical applications of LLMs [3], the addition of human verification steps at both the input and output stages for LLM-guided generation of clinical texts [22], and the implementation of self-questioning—a novel prompting strategy that encourages prompt iteration to improve accuracy in a medical context—are all steps in the correct direction. Comprehensive frameworks that include the use of diverse training data sources and continuous evaluation of bias, such as those proposed by the World Economic Forum and the Coalition for Health AI, can provide useful guardrails as new proposals for ethical validation and have been tested [23,24]. Furthermore, ensuring that physicians are actively involved in the development and evaluation of LLMs for health care is essential in keeping with a physician-led approach. Strategies such as these are key in navigating the ethics of empathy and equity in the development of novel clinical technologies.

It is essential to approach the ethical conundrums of LLM adoption in clinical care with a balanced perspective. LLMs that were built on data with inherent systemic biases must be implemented strategically into health care through a justice-oriented innovation lens to advance health equity. To keep pace with the accelerated adoption of LLMs in the clinic, ethical evaluations should be conducted together with an evaluation of use case efficacy to ensure both efficient and ethical health care. A complete assessment of the risks and benefits associated with this technology—an admittedly challenging task—may remain elusive if not tested in real-world settings. Clinical use cases of LLMs are already being tested; delaying collaboration among all stakeholders, including health care professionals, ethicists, AI researchers, and (crucially) patients, will only delay the discovery of potential harms. Real-world pilots, therefore, should be deployed alongside regular monitoring, oversight, and feedback from all parties. As we collectively seek to make full use of this exciting new technology, we must keep empathy and equity at the forefront of our minds.

**Table 1:** Approaches to the incorporation of large language models (LLMs) in clinical care.

Approach	Primary motivation	Impact on empathy and health equity
LLM-led clinical care or patient-facing LLMs	Advancement-driven: incorporation of new and sophisticated technologies mainly aimed at improving efficiency	Perpetuates and exacerbates inequities and biases on which it was built, making it. detrimental to achieving health equity Replaces human empathy with artificial empathy, which threatens patient dignity
Physician-led LLM incorporation in clinical care	Holistic, equitable, and empathetic health care delivery	Early recognition of ways in which models perpetuate inequity and appropriate measures to prevent this Opportunity to actively leverage LLMs to mitigate existing inequities Use of LLMs as tools in a physician’s toolkit allows more time to engage in empathetic dialogue with patients

**Table 2:** Potential proactive measures for promoting equitable incorporation of large language models (LLMs) into clinical care.

Stakeholder	Examples of proactive measures
Regulatory bodies	Development of robust regulations for software as a medical device that ensure appropriate strategies for (1) continuous evaluation of evolving technology and (2) assessment of use cases that have significant impact in health care given the broad capabilities of LLMs
Professional societies	Development and continuous updates of guidelines for equitable use of LLMs in health care Allocation of grant funding toward projects that aim to use LLMs to ameliorate inequities
Journals	Prioritizing publications that focus on (1) novel methods of leveraging LLMs for equitable care delivery and (2) comparisons of use cases of LLMs for equitable care delivery
Software developers and industry	Collaboration with health care workers on model improvement strategies that improve health equity

## Abbreviations

AI: artificial intelligence
EHR: electronic health record
LLM: large language model
